# Doubly curved nanofiber-reinforced optically transparent composites

**DOI:** 10.1038/srep16421

**Published:** 2015-11-10

**Authors:** Md. Iftekhar Shams, Hiroyuki Yano

**Affiliations:** 1 Research Institute for Sustainable Humanosphere, Kyoto University, Uji, Gokasho, Kyoto 611-0011, Japan

## Abstract

Doubly curved nanofiber-reinforced optically transparent composites with low thermal expansion of 15 ppm/k are prepared by hot pressing vacuum-filtered Pickering emulsions of hydrophobic acrylic resin monomer, hydrophilic chitin nanofibers and water. The coalescence of acrylic monomer droplets in the emulsion is prevented by the chitin nanofibers network. This transparent composite has 3D shape moldability, making it attractive for optical precision parts.

Glass is the most common transparent material, and is used extensively in different industries, particularly automobiles and electronics, because of its low thermal expansion, hardness, and abrasion resistance. However, because of its high density and difficulty in forming complex shapes, there is a strong demand to find alternatives to glass with lower weight, low thermal expansion and high mechanical strength. Plastic is an attractive candidate because it can possess reasonable moldability and optical properties. However, the high coefficient of thermal expansion and low rigidity and strength of plastics hinder their use in certain applications. One efficient route to improve the thermal expansion and mechanical properties of plastics while minimizing loss of transparency is to use fillers with a diameter much smaller than the wavelength of visible light^1^. Nanofibers are believed to have the potential to substantially improve the mechanical properties of plastics because their large interfacial area enables an applied load to be transferred through the filler/matrix interface while maintaining transparency^2^,^3^.

As next-generation environmentally friendly materials, bio-based nanofillers, especially cellulose and chitin nanofibers with a width of 10–20 nm, have been attracting considerable interest because of the development of new efficient isolation processes^4^,^5^,^6^,^7^. The elastic modulus of the crystalline regions of cellulose I is 138 GPa^8^,^9^, and tensile strength of nanofibrillated cellulose from wood is 1.6–3 GPa^10^. The uniform dispersion of cellulose or chitin nanofibers results in optically transparent composites because the width of these nanofibers is less than one-tenth of the optical wavelength (400–800 nm), which can eliminate light scattering^11^,^12^,^13^,^14^. Compared with cellulose nanofibers, chitin nanofibers are less hydrophilic, which leads to better compatibility with hydrophobic resins, and exhibit higher transparency^15^.

The major obstacle in reinforcing optically transparent matrices with cellulose and chitin nanofibers has been the difficulty in attaining well-dispersed nanofibers in a hydrophobic matrix. When cellulose nanofibers are directly mixed with a hydrophobic matrix resin, satisfactory nanofibers dispersion is seldom achieved because the nanofibers tend to aggregate. Various processing strategies have encountered challenges caused by nanofiber aggregation. For example, cellulose nanofibers hydrophobized by acetylation were mixed carefully in acetone with amorphous polylactic acid, but the film obtained was opaque^16^. Meanwhile, direct addition of a cellulose nanofiber suspension into a polymer melt in an extruder resulted in fiber agglomeration^17^.

Therefore, to prepare optically transparent nanocomposites, cellulose or chitin nanofibers are first dispersed in water and then filtered to form nanofiber sheets. The sheets are then impregnated with a curable resin such as epoxy or acrylic resin, which replace the air interstices and yield transparent nanocomposites with low thermal expansion^11^,^12^,^13^,^14^. However, the formation of interfiber bonding that commonly occurs between both cellulose and chitin nanofibers during sheet preparation makes it difficult to prepare 3D complex shapes, which are desired for next-generation optically transparent precision parts.

An emulsion is a system of dispersed droplets of one immiscible liquid in another stabilized by emulsifiers, while Pickering emulsions are solely stabilized by small particles^18^. Pickering emulsions are extremely stable because of the irreversible adsorption of particles at the interface between the dispersed and continuous phases^19^,^20^. Nanofibrillated cellulose, chitin, and bacteria celluloses are good bio-based candidates to stabilize emulsions because they can form a strong network that prevents coalescence of the emulsion droplets^21^,^22^,^23^,^24^,^25^,^26^,^27^. Novel renewable nanocomposite foams made from acrylated epoxidized soybean oil and hydrophobized bacterial cellulose nanofibrils have been produced using Pickering emulsion templating^28^. In this study, we introduce a simple approach to fabricate doubly curved optically transparent chitin acrylic resin nanocomposites by Pickering emulsification. To our knowledge, this is the first report for fabricating double curved nanofiber-reinforced composite. The tiny acrylic resin droplets that are generated in aqueous suspensions of chitin nanofibers through emulsification are stabilized by the chitin nanofiber network in water to produce unique 3D nanocomposites after filtration. Notably, the composites prepared by this simple process consisting of emulsification, filtering, drying and hot pressing exhibit low thermal expansion at low fiber content compared with that of acrylic resin-impregnated nanofiber sheets.

## Methods

### Preparation of chitin nanofibers

Commercial α-chitin powder from crab shells was used as a starting material. Dry chitin powder was dispersed in water at a concentration of 0.4 wt% to form slurry. Several drops of acetic acid were added to the chitin slurry to adjust the pH to 3–4 and facilitate fibrillation. The slurry was crushed roughly with a domestic blender, stirred for 1 h to remove air bubbles and then passed through a high-pressure homogenizer (Star Burst, Sugino Machine Co. Ltd.) 30 times.

### Emulsification

An aqueous suspension containing a mixture of acrylic resin and chitin nanofibers (3 wt%) was prepared by mixing a suspension of chitin nanofibers (0.5 g) in water (166 g) with acrylic resin (4.5 g, ABPE10, refractive index 1.513, Shin-Nakamura Chemical Co. Ltd.) for 60 min with vigorous stirring. The suspension was then subjected to mechanical agitation (Vita-Mix Blender, Osaka Chem. Co. Ltd.) for 15 min. The resulting emulsion was diluted to a concentration of approximately 0.1% w/w using distilled water and observed using an optical microscope (VHX-200, Keyence). The slurry was also freeze-dried at −40 °C, coated with Pt (thickness of ca.2 nm) in an ion-sputter coater (JFC-1600, JEOL Ltd.), and observed by FE-SEM (JSM-6700F JEOL).

To avoid multiple scattering effects, the emulsion samples were diluted to a concentration of approximately 0.1% w/w with distilled water and stirred gently with a spatula until completely dispersed. Samples were analyzed by a laser scattering particle size distribution analyzer (LA-950V2, Horiba Ltd.).

### Nanocomposite fabrication

The emulsion was vacuum filtered through a metal mesh (300 mesh) and polytetrafluoroethylene membrane filter (0.1 μm, Advantech Co. Ltd.) to produce a wet thin sheet with a diameter of 40 mm. The sheets were dried at 50 °C in a convection oven for 4 h. The fracture surface of the sheet was observed by FE-SEM. Transmission electron microscopy (TEM) observation was carried out using a JEOL JEM1230 instrument at an accelerating voltage of 120 kV.

Acrylic resin-chitin nanofiber sheets were placed between two glass plates and mechanically pressed at 110 °C for 10 min under different pressures. The samples were cooled to ambient temperature under pressure and then immediately cured using UV curing equipment (20 J/cm^2^, F300S UV lamp system and LC6 benchtop conveyor, Fusion UV Systems, Inc.) to obtain the composite sheets. The fiber content of the composites was calculated based on the original weight of fibers and the final oven-dried weight of the composites. The fiber content of the composites was 14%.

### Composite Characterization

Regular light transmittance of samples was measured at wavelengths of 200 to 800 nm using a UV-visible spectrometer (U-4100, Hitachi High-Tech. Co.) with an integrating sphere with a diameter of 60 mm. Regular transmittance was measured by placing the sample 25 cm from the entrance port of the integrating sphere. CTE values were measured at tensile mode by a thermomechanical analyzer (TMA/SS6100, SII Nanotechnology Inc.). Samples had a length of 25 mm, width of 3 mm, thickness of 150–160 μm and span of 20 mm via a vertically adjustable quartz glass probe. The specimen is held between two clamps and then suspended in the measuring device. To prevent the specimen from twisting or curling, it is loaded with a slight tensile force. Measurements were carried out three times during elongation at a heating rate of 5 °C/min in a nitrogen atmosphere at a load of 3 g. CTE were determined as mean values at 20–150 °C in the second run.

## Results and Discussion

**Figure 1:**
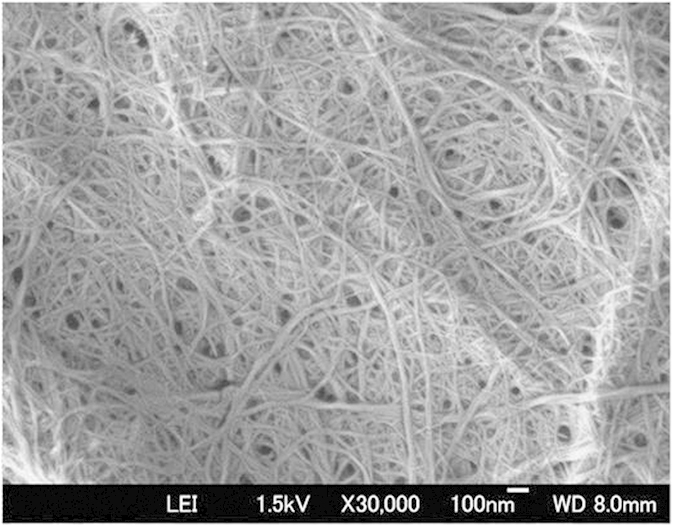
FE-SEM image of uniform chitin nanofibers obtained after high pressure homogenization.

**Figure 2:**
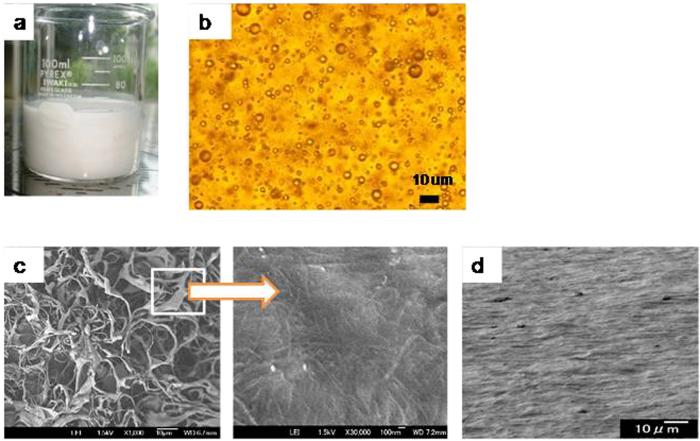
(**a**) Photograph of an acrylic resin-chitin nanofiber suspension obtained after stirring and mechanical agitation. (**b**) Optical microscope image of a resin-water emulsion stabilized by nanofibers. (**c**) FE-SEM images of a freeze-dried emulsified mat (left, ×1000, and right, ×30,000). (**d**) Fracture surface of an acrylic resin-chitin nanofiber sheet after drying.

Figure 1 shows a field-emission scanning electron microscope (FE-SEM) image of chitin nanofibers after homogenization. Chitin nanofibers with a diameter of 10 to 20 nm and large surface area were obtained. Effective dispersion of the chitin nanofibers within the matrix is critical to fully utilize the reinforcing properties of the chitin nanofibers. To achieve this, an aqueous suspension containing 3 wt% of a mixture of acrylic resin and chitin nanofibers was prepared by dispersing chitin nanofibers (0.5 g) in water (166 g) and then adding acrylic resin (4.5 g) with stirring, followed by mechanical agitation. The fiber content in the solid content was 10%. Vigorous agitation of the suspension of chitin nanofibers and acrylic resin led to the formation of resin droplets dispersed in water (a Pickering emulsion), which appeared as a white viscous emulsion (Fig. 2a) that was stable for months. An optical microscope image of the emulsion clearly indicated that the dispersion of nanofibers influenced its stability (Fig. 2b). The average resin droplet size in the emulsion determined by dynamic light scattering was less than 10 μm. A FE-SEM image of the freeze-dried emulsified mat (Fig. 2c, left) shows its porous structure of ribbon-like materials. A high-magnification image (Fig. 2c, right) revealed that in the ribbon-like materials, the chitin nanofibers formed networks that were embedded uniformly within the acrylic resin.

Because the resin matrix contained a uniform distribution of nanofibers, the freeze-dried mat was compressed at 15 MPa and 110 °C to remove trapped air. However, this did not result in a transparent composite. Although the resin-nanofiber mat was deformed under loading, the trapped air was not fully removed because of the rigid 3D nanofiber structure. Therefore, we used filtration to form composite sheets. When the mixture was slowly filtered through a 0.1-μm filter membrane to obtain a wet sheet, a mixture of acrylic monomer and chitin nanofibers stayed on the filter. Because the nanofibers were concentrated during filtration to form a dense network, a large amount of hydrophobic acrylic resin was trapped in the hydrophilic nanofiber network and retained in the sheet. The fiber content of the dried composites was 14%. Figure 2d shows a FE-SEM image of the fracture surface of a resin-nanofiber sheet after drying. The cross-section of the composites clearly possesses lamellar structure, indicating that the nanofibers were concentrated by filtration to form an in-plane network. The resin-nanofiber sheet obtained was opaque. However, when this sheet was mechanically pressed at 110 °C, it became transparent.

**Figure 3:**
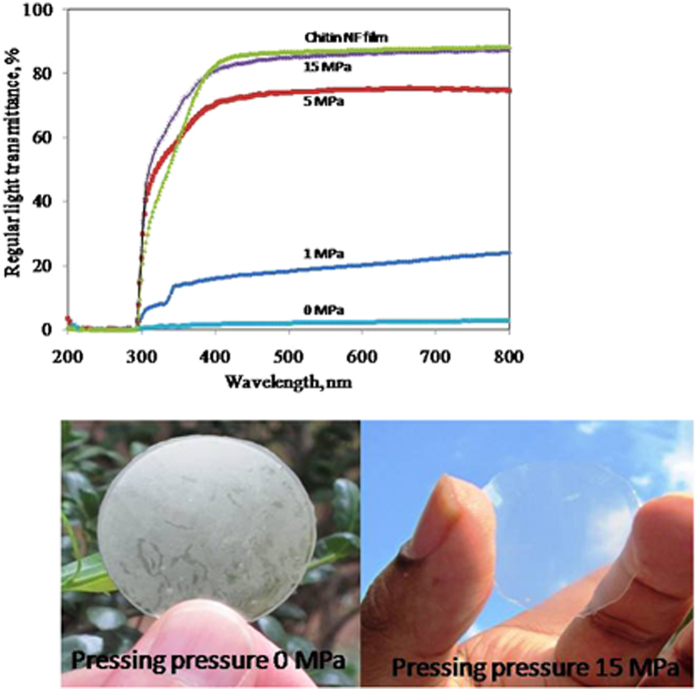
Changes in optical transparency of molded acrylic resin-chitin nanofiber sheets upon mechanical pressing. Molded sheet thickness: 150–160 μm and acrylic resin-impregnated nanofiber sheet thickness: 110 μm.

The success of this process was confirmed by regular light transmittance measurements. The regular transmittances of the sheets before and after pressing at different pressures are compared in Fig. 3. When a sheet was heated at 110 °C without pressing, its regular transmittance at 600 nm was almost 0%. However, the transmittance of the sheets increased with the pressure used during hot pressing. The regular transmittance of the composite pressed at 1 MPa was 20%, but it reached 86% at 600 nm for that compressed at 15 MPa (Fig. 3). Interestingly, the difference between total and regular optical transmittance for the acrylic resin-impregnated nanofiber sheets was small (Fig. 3 and Table 1). For example, the linear light transmittance of acrylic resin-impregnated nanofiber sheets was 87.3% whereas the developed chitin nanocomposites at 15 MPa were 86.1%.

Another notable feature of the molded transparent films is their low coefficient of thermal expansion (CTE), as shown in Table 1. The addition of 14 wt% chitin nanofibers to the acrylic resin markedly decreased its CTE from 213 to 15.1 ppm/K. The random distribution of in-plane oriented nanofibers (Fig. 2d) apparently leads to the very low CTE of the corresponding composites. Additionally, fiber-fiber and fiber-resin interactions might be further amplified by compression, as indicated by the decrease in CTE with increasing linear light transmittance, as shown in Table 1. The resin-nanofiber composites compressed at 0 MPa showed significantly different CTE value in contrast with the other composites obtained by pressing pressure. Through pressing pressure of 5 MPa, the CTE decreased from 19.7 ppm/K to 16.2 ppm/K. However, it can readily be seen that above pressing pressure of 5 MPa, the CTE values did not differ (Table 1). Notably, the CTE obtained for the acrylic resin-nanofiber sheets following 15-MPa compression (15.8 ppm/K) is comparable to that of acrylic resin-impregnated nanofiber sheets with a higher chitin fiber content of 35–40%.

**Figure 4:**
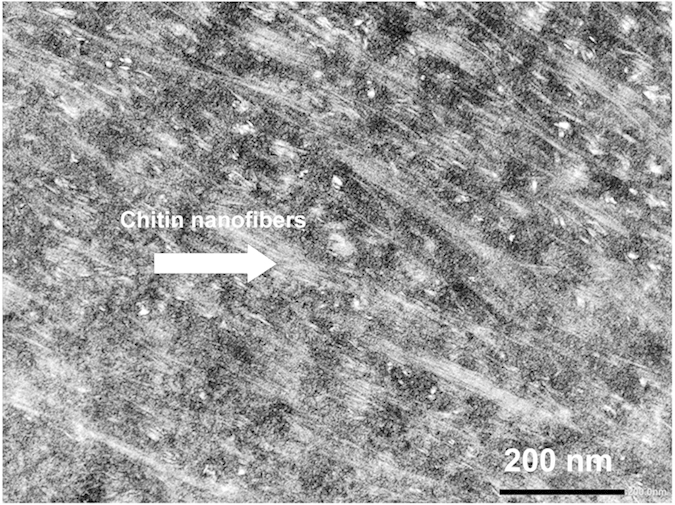
Transmission electron microscopy (TEM) image of acrylic resin chitin nanofiber sheet.

The mechanism responsible for the high level of transparency obtained by using a Pickering emulsion to form these nanocomposites can be explained as follows. Once the water was removed by vacuum filtration, the resin droplets that were covered with chitin nanofibers formed a concentrated network that trapped the resin droplets. During drying at 50 °C, the resin softened and droplets flattened, which is consistent with the observed lamellar structure (Fig. 2d). From the TEM image (shown in Fig. 4) it is clearly seen that most of chitin nanofibers were distributed uniformly in acrylic resin. Acrylic resin penetrated well among chitin nanofibers and adhered between nanofiber layers. Subsequent mechanical pressing at high temperature (110 °C) removed the air and further enhanced the low-viscosity resin to penetrate into the nanofibers, so the sheets became transparent.

**Figure 5:**
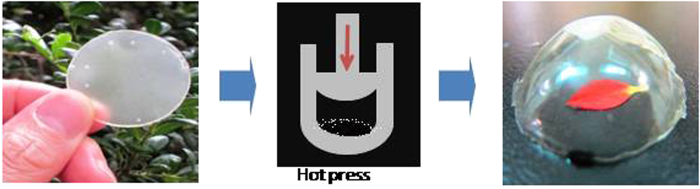
Preparation of molded complex 3D optically transparent chitin nanocomposites.

The resin-nanofiber sheet was very soft and could be enlarged during hot pressing because of the plasticity of the resin. This allowed us to prepare a 3D die specifically for molding and hot pressing the sheet, as shown in Fig. 5. The samples were easily molded while retaining transparency. This strongly indicates that the acrylic resin may prevent, or at least severely limit, the formation of strong hydrogen bonds between the chitin nanofibers. This is the first example of a doubly curved optically transparent nanofiber-reinforced composite.

## Conclusions

In summary, a new process to fabricate doubly curved optically transparent nanocomposites with low thermal expansion has been established. Emulsions of chitin nanofibers and acrylic resin are stabilized by the hydrophilic and high-specific-surface-area chitin nanofiber networks preventing the coalescence of tiny emulsion resin droplets. The chitin nanofibers and hot pressing at high pressure increase the transparency and decrease the CTE of the composites. This composite will be useful in 3D optical precision parts such as optical lenses, as flexible optically transparent substrates for display and solar cells indented with tiny, precise lenses on their surface, and transparent 3D engineering parts.

## Additional Information

**How to cite this article**: Shams, M. I. and Yano, H. Doubly curved nanofiber-reinforced optically transparent composites. *Sci. Rep.* **5**, 16421; doi: 10.1038/srep16421 (2015).

## Figures and Tables

**Table 1:** Influence of processing technique on the total and regular transmittance and thermal expansion of chitin nanocomposites* 15.

	Fiber content (%)	Light transmittance (%, at 600 nm)		CTE (ppm/K)
		Linear	Total	
Acrylic resin	0	91.3	92.0	213.0 (2.12)
Molding composites at 0 MPa	14	2.2	84.8	19.7 (0.98)
Molding composites at 5 MPa	14	74.8	88.7	16.2 (1.68)
Molding composites at 10 MPa	14	83.2	88.8	16.2 (0.91)
Molding composites at 15 MPa	14	86.1	90.4	15.1 (0.88)
Nanofiber sheet impregnated composites*	35–40	87.3	90.5	15.8

Values are average of four samples. Values presented in parenthesis are standard deviation.
